# Visceral Obesity Is Associated with Shorter Progression-Free Survival in Well-Differentiated Gastro-Entero-Pancreatic Neuroendocrine Neoplasia

**DOI:** 10.3390/jcm11206026

**Published:** 2022-10-12

**Authors:** Ana P. Santos, Jessica Rodrigues, Rui Henrique, M. Helena Cardoso, Mariana P. Monteiro

**Affiliations:** 1 Department of Endocrinology, Portuguese Oncology Institute of Porto (IPO Porto), Porto Comprehensive Cancer Centre (P.CCC), 4200-072 Porto, Portugal; 2 Precancerous Lesions and Early Cancer Management Group, Research Center of IPO Porto (CI-IPOP), RISE@CI-IPO (Health Research Network), Portuguese Oncology Institute of Porto (IPO Porto), Porto Comprehensive Cancer Centre (P.CCC), 4200-072 Porto, Portugal; 3 Cancer Epidemiology Group, Research Center of IPO Porto (CI-IPOP), RISE@CI-IPOP (Health Research Network), Portuguese Oncology Institute of Porto (IPO Porto), Porto Comprehensive Cancer Centre (P.CCC), 4200-072 Porto, Portugal; 4 Department of Pathology, Portuguese Oncology Institute of Porto (IPO Porto), Porto Comprehensive Cancer Centre (P.CCC), 4200-072 Porto, Portugal; 5 Cancer Biology and Epigenetics Group, Research Centre of IPO Porto (CI-IPOP), RISE@CI-IPOP (Health Research Network), Portuguese Oncology Institute of Porto (IPO Porto), Porto Comprehensive Cancer Centre (P.CCC), 4200-072 Porto, Portugal; 6 Department of Pathology and Molecular Immunology, ICBAS—School of Medicine and Biomedical Sciences, University of Porto, 4050-313 Porto, Portugal; 7 Department of Endocrinology, Centro Hospitalar Universitário do Porto (CHUPorto), 4099-001 Porto, Portugal; 8 UMIB—Unit for Multidisciplinary Research in Biomedicine, ICBAS—School of Medicine and Biomedical Sciences, University of Porto, 4050-313 Porto, Portugal; 9 ITR—Laboratory for Integrative and Translational Research in Population Health, 4200-450 Porto, Portugal

**Keywords:** gastro-entero-pancreatic neuroendocrine neoplasia, metabolic syndrome, visceral obesity, overall survival, progression-free survival

## Abstract

The association of well-differentiated gastro-entero-pancreatic neuroendocrine neoplasia (WD GEP-NEN) with metabolic syndrome (MetS), abdominal obesity, and fasting glucose abnormalities was recently described. However, whether obesity and metabolic syndrome risk factors are associated with GEP-NEN adverse outcomes and the poorer prognosis was unknown. The present study aimed to evaluate whether the presence of MetS or any of its individual components at WD GEP-NEN diagnosis influenced disease outcomes. A cohort of patients with non-localized WD GEP-NETs (*n* = 81), was classified according to the primary tumor site (gastrointestinal or pancreatic), pathological grading (G1 (Ki67 ≤ 2%) and G2 (3% ≤ Ki67 ≤ 20%) (WHO 2010)), disease extension (loco-regional or metastatic disease), presence of hormonal secretion syndrome (functioning or non-functioning), and evaluated for the presence of MetS criteria at diagnosis. MetS was present in 48 (59.3%) patients. During a median follow-up of 95.0 months (16.8–262.5), 18 patients died of the disease (10 with MetS vs. 8 without MetS). Overall survival (OS) at 5 years was 87.1% (95% CI: 73.6–94.0) for MetS and 90.9% (95% CI: 74.4–97.0) for non-Mets group, while OS at 10 years was 72.5% (95% CI: 55.3–84.0) for MetS, and 76.4% (95% CI: 53.6–89.0) for non-MetS group. Progression-Free Survival (PFS) at 5 years was 45.9% (95% CI: 30.8–59.8) for MetS and 40.0% (95% CI: 21.3–58.1) for non-MetS group, and PFS at 10 years was 18.1% (95% CI: 7.0–33.5) for MetS and 24.4% (95% CI: 9.0–43.7) for non-MetS group. Waist circumference (WC), a surrogate measure for visceral obesity, was associated with significantly shorter PFS (HR = 1.03; 95% CI: 1.01–1.06), although did not influence OS (HR = 1.01; 95% CI: 0.97–1.06). The findings of this study reinforce a potential link between visceral obesity and GEP-NEN and further suggest that obesity could influence disease prognosis.

## 1. Introduction

Gastro-entero-pancreatic neuroendocrine neoplasia (GEP-NEN) was considered a rare entity before the 460% increase in incidence observed over the past four decades [1]. The association between environmental factors and cancer is well established for several neoplasias, although with different levels of evidence according to the type of tumor considered [2,3,4,5]. Unhealthy dietary patterns, sedentary life, obesity, and diabetes are the modifiable risk factors (RFs) that were recognized to contribute to nearly 30% of new cancer diagnoses [6,7,8,9,10]. Less well-characterized is the influence of the above-mentioned RFs on cancer outcomes, although the association with a higher recurrence and mortality rate has been reported for colorectal, breast, uterus, stomach, prostate, and pancreas, among other neoplasias [11,12,13,14,15,16]. Although MetS and MetS individual components, namely abdominal obesity and abnormal fasting plasma glucose (FPG), have been recently described to be associated with well-differentiated GEP-NEN by our group [17], data on how obesity, diabetes, and other metabolic syndrome RF’s influence GEP-NEN’s prognosis was lacking.

The aim of the current study was to evaluate whether the presence of obesity, MetS and MetS individual components, at the time of WD GEP-NEN diagnosis influenced overall survival (OS) and progression-free survival (PFS) from the first treatment intervention in individuals with non-localized disease.

## 2. Materials and Methods

Patients with confirmed WD GEP-NETs were recruited from the Endocrine Tumors Clinic at a single national tertiary referral center for oncologic diseases (Portuguese Oncology Institute of Porto; IPOFG, Porto, Portugal). The criteria for inclusion were WD GEP-NEN diagnosis confirmed by histopathology and/or PET-68Ga-DOTA-NOC. Patients under 18 years old at diagnosis, harboring familial GEP-NEN, neuroendocrine carcinoma (NEC), and type 1 gastric endocrine neoplasia (T1-GEN) were excluded from the study, as these tumors have a distinct biological behavior and/or established etiologies [18].

Among patients with WD GEP-NEN, who consented to participate in the study (*n* = 136), those who did not fulfill the inclusion criteria or had insufficient data for analysis were excluded (*n* = 55). All the remaining eligible patients were included for statistical analysis (*n* = 81). Tumors were classified according to primary tumor site: gastrointestinal (GI-NEN) or pancreatic (pNEN); hormonal secretion: functioning or non-functioning (F or non-F); pathological WHO 2010 grade: G1 (<2 mitotic count; Ki-67 ≤ 2) and G2 (2–20 mitotic count; 3% ≤ Ki67 ≤ 20%) and disease extension (loco-regional or metastatic disease) [19]. Non-localized disease extension was categorized as locoregional or metastatic, to enable the grouping of WD GEP-NENs, since ENETS staging categories diverge according to the primary tumor site. Patients with insufficient data to allow grading were classified as WD GEP-NEN only if found to express somatostatin receptors on ^68^Ga-DOTA-NOC-PET/CT (*n* = 2). Patients with WD GEP-NEN-presenting metastatic disease and carcinoid syndrome without any visible pancreatic or thoracic lesions on imaging studies were assumed as having midgut primary tumors (*n* = 2). No insulinoma or rare functional pancreatic NEN presenting with hyperglycemia, such as glucagonoma, VIPoma, or somatostatinoma, were included in this patient series [20].

Patients with WD GEP-NENs were assessed for body mass index (BMI) class [21], fasting plasma glucose (FPG) category [22], and the presence of MetS diagnostic criteria or any individual MetS component [23].

Data for analysis were collected during face-to-face patient interviews, to assess past medical history of type 2 diabetes (T2D), hypertension, dyslipidemia, ongoing medications, and family history of T2D. Anthropometric parameters, such as height, weight, waist circumference (WC), and blood pressure (BP), and biochemical data, including FPG and lipid profile, were evaluated after blood sampling at our institution for treatment-naïve patients, or retrospectively through data-files recollection of parameters before initiation of any treatment intervention at other healthcare institutions, whenever the patient was already under treatment by referral to our center.

Patients were classified into three categories according to BMI: normal weight (BMI < 25 kg/m^2^), overweight (25 kg/m^2^ ≤ BMI < 30 kg/m^2^), or obese (BMI ≥ 30 kg/m^2^) [21]. Patients were also classified according to FPG levels: euglycemic (NG; FPG < 100 mg/dL), impaired fasting glucose (IFG; 100 mg/dL ≤ FPG < 126 mg/dL), or T2D (FPG ≥ 126 mg/dL) [22]. MetS was classified according to the Joint Interim Statement (JIS) of IDFTFEP (International Diabetes Federation Task Force on Epidemiology and Prevention)/NHLBI (National Heart, Lung, and Blood Institute)/AHA (American Heart Association)/WHF (World Heart Federation)/IAS (International Atherosclerosis Society)/IASO (International Association for the Study of Obesity) criteria [23]: WC ≥ 88 cm (female) or 102 cm (male); systolic BP ≥ 130 or diastolic BP ≥ 85 mmHg or previous history of high BP or under BP-lowering medication; HDL-cholesterol (HDL-c) < 40 mg/dL (male) or ≤ 50 mg/dL (female) or drug treatment to reduce HDL-c; triglycerides (TG) ≥ 150 mg/dL or on triglyceride-lowering drugs; FPG ≥ 100 mg/dL or ongoing treatment with glucose-lowering drugs.

OS was defined as the time in months between the date of first treatment intervention and the date of last contact or death. PFS was defined as the time in months between the date of the first treatment and the date of first progression detection, date of the last contact, or death.

Statistical analysis was performed using R software v4.0.5 (R Foundation for Statistical Computing, Vienna, Austria). Categorical variables were summarized as frequencies and percentages. Continuous variables were presented as median, minimum, and maximum. Chi-square or Fisher’s exact tests were used to evaluate the association between two categorical variables. Medians between groups were compared using the Mann–Whitney or Kruskal–Wallis test. OS, PFS, and the corresponding 95% confidence intervals (CIs) were estimated using the Kaplan–Meier method. Survival between groups was compared by the log-rank test. Cox proportional-hazards regression analysis was used to compute hazard ratios (HR) with the corresponding 95% CIs. For the Cox proportional-hazards regression analysis, univariate models were performed, and each variable was considered separately. Then, the variables that were considered statistically significant in the univariate models were retained for the multivariable model. The final multivariable model contains the variables that were statistically significant. All tests of statistical significance were two-sided; a *p* value < 0.05 was considered significant.

## 3. Results

### 3.1. Baseline Characteristics of Patient Population with WD GEP-NEN

Patients with WD GEP-NEN (*n* = 81) were divided into two groups, according to baseline characteristics considering the absence (*n* = 33; 40.7%) or presence of MetS (*n* = 48; 59.3%) at diagnosis (Table 1). Patients in the MetS group were older (63 years for MetS vs. 53 years for non-MetS; *p* = 0.002), but there was no significant difference in gender distribution (*p* = 0.262). As expected, patients with MetS group had higher median BMI, WC, SBP, DBP, triglycerides and FPG levels than non-MetS group.

When comparing patients with or without MetS, there were no differences in the primary tumor site, presence of hormonal secretion syndrome, or metastatic disease. G1 tumors were more frequent in patients with MetS (64.6%) than non-MetS (51.5%), although this difference was not statistically significant (*p* = 0.241). There were also no statistically significant differences in follow-up time for OS (101.7 months for non-MetS vs. 84.7 months for. MetS; *p* = 0.204), nor in follow-up time for PFS (50.6 months for non-MetS vs. 50.7 months for MetS; *p* = 0.966), between the two groups.

### 3.2. Overall Survival and Progression-Free Survival at 5 and 10 Years according to the Presence of Metabolic Syndrome

No statistically significant differences were found in OS and PFS rates at 5 and 10 years between patients with MetS and without MetS (Table 2). The OS rate for patients with MetS was 87.1% (95% CI: 73.6–94.0) at 5 years, whereas the rate was 90.9% (95% CI: 74.4–97.0) for patients without MetS. The OS rate at 10 years was 72.5% (95% CI: 55.3–84.0) for patients with MetS and 76.4% (95% CI: 53.6–89.0) for patients without MetS. ThPFS rate for patients with MetS was 45.9% (95% CI: 30.8–59.8) at 5 years, whereas the rate was 40.0% (95% CI: 21.3–58.1) for patients without MetS. The PFS rate at 10 years was 18.1% (95% CI: 7.0–33.5) for patients with MetS and 24.4% (95% CI: 9.0–43.7) for patients without MetS.

There were no significant differences in the presence of MetS individual components namely, waist circumference, hypertension, elevated triglycerides, low HDL-c, and elevated FPG (Table 2) in OS and PFS at 5 and 10 years.

### 3.3. Median Overall Survival and Progression-Free Survival of Patients with WD GEP-NEN according to the Presence of MetS

**Figure 1:**
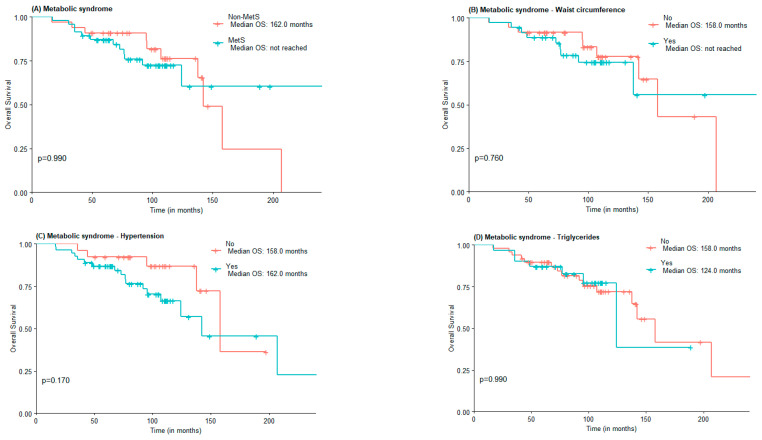

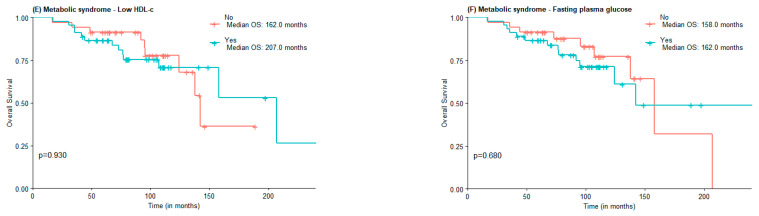
OS according to the presence of Metabolic Syndrome (**A**) and Metabolic Syndrome components waist circumference (**B**), hypertension (**C**), high triglycerides (**D**), low HDL-c (**E**), and Fasting plasma glucose (**F**).

The median OS was 142.0 months for the non-MetS group and was not reached for the MetS one (Figure 1A). For abdominal obesity measured by WC, the median OS was 158.0 months for patients without abdominal obesity and not reached for patients with abdominal obesity (Figure 1B). The median OS was lower in the group with MetS criteria for hypertension vs. non-MetS group (142.0 vs. 158 months) (Figure 1C). The median OS for elevated triglycerides and FPG was lower in the MetS group, although not statistically significant (124 vs. 158 months and 142 vs. 158 months, respectively). Interestingly, the median OS of patients with low HDL-c was higher when compared to those of patients with normal or elevated HDL-c (207.0 months vs. 142.0 months), in the low HDL-c group there was also a greater proportion of patients under statin treatment (Figure 1E).

**Figure 2:**
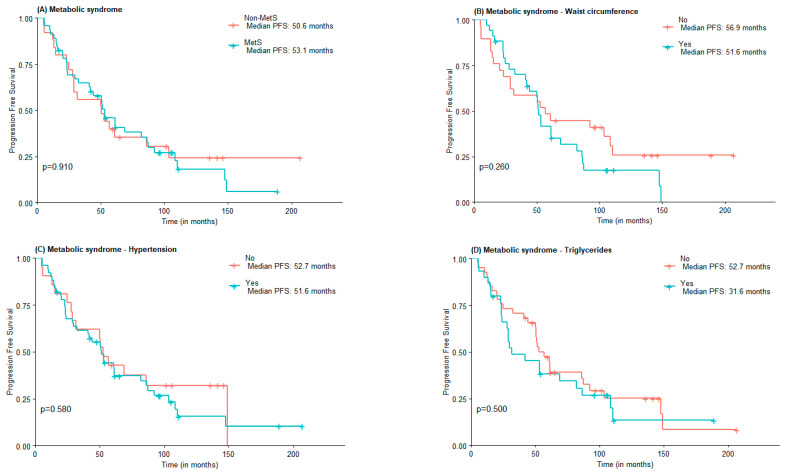

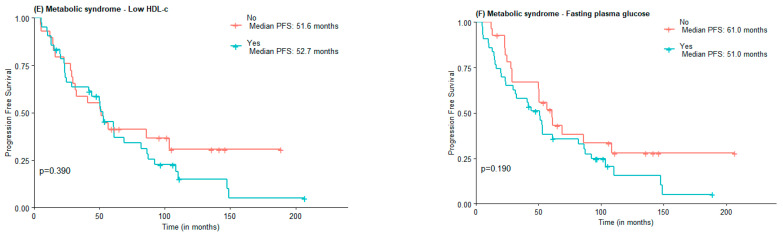
PFS according to the presence of Metabolic Syndrome (**A**) and Metabolic Syndrome components waist circumference (**B**), hypertension (**C**), high triglycerides (**D**), low HDL-c (**E**), and Fasting plasma glucose (**F**).

Regarding PFS, no statistically significant differences were found between MetS and non-MetS groups (53.1 vs. 50.6 months), nor concerning MetS components elevated WC (51.6 vs. 56.9 months), hypertension (51.6 vs. 52.7 months), and HDL-c (52.76 vs. 51.6 months). Subjects with elevated triglycerides and FPG had lower PFS compared to those with normal triglycerides and FPG, although not statistically significant (31.6 vs. 52.7 months and 51.0 vs. 61.0 months) (Figure 2).

### 3.4. Influence of Tumor Pathological Features on Patient Outcomes

When considering all the characteristics individually, WHO tumor grade was the only characteristic that influenced OS, as patients diagnosed with grade 2 tumors had shorter OS than patients diagnosed with grade 1 tumors (HR = 3.84; 95% CI: 1.41–10.50). PFS was influenced by WHO grading (patients who presented grade 2 tumors had a shorter PFS than patients diagnosed with grade 1 (HR = 2.34; 95% CI: 1.30–4.22)), stage (patients diagnosed with metastatic disease had a shorter PFS when compared to patients diagnosed with locoregional disease; HR = 3.11; 95% CI: 1.33–7.30), and waist circumference (patients with elevated waist circumference had a shorter PFS than patients with normal waist circumference; HR = 1.03; 95% CI: 1.01–1.06).

When considering a multivariable model only WHO grading (HR = 2.17; 95% CI: 1.15–4.11) and waist circumference (HR = 1.03; 95% CI: 1.01–1.06) influenced survival outcomes. Table 3 presents the hazard ratios for OS and PFS according to patient and tumor characteristics.

## 4. Discussion

To the best of our knowledge, this is one of the first studies addressing the influence of obesity and MetS on GEP-NEN outcomes and the first that includes abdominal obesity. In our patient cohort with WD GEP-NEN, using waist circumference as a surrogate marker for visceral obesity at diagnosis, was shown to have a prognostic value for PFS in patients with non-localized WD GEP-NEN (HR = 1.01; 95% CI: 1.01–1.06), although not affecting OS. Additionally, this study also confirmed previous findings that intrinsic tumor factors, namely WHO grade G2 influences OS (HR = 3.84; 95% CI: 1.41–10.50) and PFS, both at univariate (HR = 2.34; 95% CI: 1.30–4.22) and multivariate (HR = 2.17; 95% CI: 1.11–4.11) analysis. Metastatic disease at diagnosis was also shown to influence PFS (HR = 3.11; 95% CI: 1.33–7.30), but not OS (HR = 3.60; 95% CI: 0.84–15.51).

The association of obesity, MetS, and T2D with cancer risk is well established. The International Agency for Research on Cancer (IARC) group and the World Cancer Research Fund/American Institute for Cancer Research (WCRF/AICR) recognized the influence of overweight and obesity as RF for 13 different types of cancers, including breast, colorectal, endometrial, esophageal adenocarcinoma, gallbladder, gastric, renal cell, liver, multiple myeloma, ovarian, pancreas, and thyroid, despite different levels of evidence applying for each type of tumor [7]. Indeed, 5.6% of all incident cancers can be attributed to the combined effects of diabetes and BMI as independent risk factors, among which high BMI was responsible for twice as many cancer cases as diabetes [24]. Additionally, there is now enough evidence to state that not only is MetS a RF for cancer, but also MetS individual components, such as visceral obesity, hyperglycemia, dyslipidemia, and hypertension, independently of BMI [25]. Moreover, a recent meta-analysis concluded that central adiposity is a stronger predictor of non-neuroendocrine tumor risk than overall body size [26].

In parallel with obesity and MetS, an exponential rise in NEN incidence has been noticed over the last forty years, which has traditionally been attributed to improved diagnostic skills and imaging techniques. However, little is known about how extrinsic RFs have influenced NEN’s burden. Our group has recently described an association between MetS and some MetS individual components, such as visceral obesity and abnormal FPG with WD GEP-NEN [17]. We have also found that patients with WD GEP-NEN and MetS had a higher risk of presenting a lower tumor grade yet disseminated disease at diagnosis, independently of primary tumor site and hormonal status [27]. In support of our previous study’s findings, a metanalysis published by Leoncini et. al. [28] concluded that obesity conferred an estimate OR = 1.37 (95% CI: 0.25–7.69; I2 = 98.5; *p* < 0.0001) for pancreatic NEN, although this conclusion was not supported by all studies included. The available data also suggested that diabetes conferred an increased risk for pancreatic NEN (OR = 2.76; 95% CI: 1.65–4.64; I2 = 55.3; *p* = 0.090), the risk being even higher for recent onset diabetes (OR = 12.80; 95% CI: 2.47–66.42; I2 = 55.3; *p* = 0.135). A recent Italian case-control study, that evaluated the influence of chronotype on WD GEP-NEN, concluded that a higher proportion of evening chronotype and unhealthy metabolic profile, along with smoking and sedentary habits, was observed in patients with GEP-NEN when compared to healthy controls [29].

Another matter of debate is the influence of obesity and metabolic diseases on cancer outcomes. In contrast to epidemiological studies seeking to identify RFs for cancer incidence, studies aimed to identify RFs for patient outcomes have been less conclusive. Additionally, these studies are often hampered by the multitude of variables to be considered, such as patient overall condition, anti-cancer treatments, and treatment side effects, as well as other ongoing treatments for chronic conditions. Notwithstanding, a recent meta-analysis was able to conclude that the survival of patients with digestive tract cancer was negatively affected by the presence of MetS [13]. MetS was also found to be associated with a higher risk of death from colorectal (HR = 3.48; 95% CI: 1.68–7.22) and breast cancer in women (HR = 11.90; 95% CI: 2.25–62.84) [14]. Moreover, overall cancer mortality was found to be higher in patients with abdominal adiposity, independently of BMI, over a mean follow-up of 9.7 years [30]. Concerning NEN, despite several pathological and molecular tumor features that have been identified to be relevant for prognosis [31,32,33,34,35], data on how extrinsic factors, such as obesity and metabolic diseases influence disease outcomes is very scarce.

A recent post-hoc analysis of the CLARINET study on the impact of diabetes and metformin at GEP-NEN PFS concluded that diabetes was not a negative prognostic factor. However, patients in the placebo group who were under metformin had longer PFS (85.7 weeks for metformin vs. 38.7 weeks for no-metformin, *p* = 0.017), an effect that was not observed in the lanreotide treatment arm [36]; a finding which favors the hypothesis that the anti-tumor effect of metformin is abrogated in the presence of mTORC1 axis inhibition by somatostatin analogues. Metformin is an anti-diabetic drug with direct action on phosphoinositide 3-kinase/serine/threonine kinase/mTor (Pi3k/Akt/mTor pathway) via Adenosine Monophosphate-Activated Protein Kinase (AMPK) suppressive activity on Mammalian Target of Rapamycin Complex 1 (mTORC1) and indirect effects on insulin secretion and insulin-like growth factor-1 (IGF-1) activity, which was also demonstrated to depict anti-cancer effects [37]. Indeed, the anti-proliferative effects of metformin in NEN, as an adjuvant treatment to conventional therapy, have been described in vitro [38] and suggested by retrospective studies [39,40,41].

The PRIME-NET Study conclusions contrast with those of CLARINET post-hoc. In the former study, patients with diabetes treated with everolimus and somatostatin analogues under metformin, had longer PFS than patients under other anti-diabetic drugs [42]. It is noteworthy that our study cohort differs from the ones of the abovementioned studies. When compared to CLARINET post-hoc, the follow-up time is longer (95.0 months vs. 24 months) and included mainly carcinoid syndrome-associated GI-tract primary tumors (55.6% midgut tumors) vs. non-functioning pNEN [36], a feature that also contrasts with the PRIME-NET study that included only patients with pNEN, some of which in the context of MEN1, while the hormonal status was not described [42]. Our study is one of the first to describe the association of MetS with functioning GI-NEN, namely midgut NEN, while most of our previous data documented the association of metabolic abnormalities with non-functioning pNEN [28]. In fact, our cohort included predominantly functioning tumors (60.5%). Moreover, our study population consisted of well-differentiated tumors (WHO 2010 NET G1 and NET G2), while CLARINET post-hoc only included tumors with Ki67 < 10% and PRIME-NET included all WHO categories, which could have biased the results. Additionally, although the percentage of patients who were overweight (40.7%) in our study was similar to the PRIME-NET and CLARINET studies, we found that almost half of our patients had abdominal obesity (45.7%), a parameter which was not assessed in the two previous studies. Diabetes was present in 23.7% of our patient cohort, vs. 38.7% in CLARINET post-hoc and 100% in PRIME-NET; while FGA was noticed in 19.8% of our cohort, and neither study evaluated intermediate hyperglycemia. Notwithstanding, our results show that elevated waist circumference was associated with shorter PFS, regardless of the fact that BMI, MetS, hypertension, dyslipidemia, and hyperglycemia were not found to be associated with poorer WD-GEP-NEN prognosis [43]. A 2021 analysis on the Surveillance, Epidemiology, and End Results (SEER) databases and linked Medicare claims (1991–2016), compared the risk for T2D in somatostatin analogues (SSA) treated vs. no-SSA treated patients, did not find significant differences, which suggests that GEP-NEN patients have a higher risk for T2D, independently of the hyperglycemic effects of treatment [44]. Similar findings were published by our group [17]. Evening chronotype also seems to influence GEP-NEN prognosis, as it showed to be associated with distant metastasis, a higher grade, and progressive disease [29].

Data on the influence of dyslipidemia on GEP-NEN prognosis is limited to few studies. Disease progression was associated with elevated triglycerides in advanced pNEN treated with everolimus, and reversed with dietary and pharmacological intervention with statins and metformin [45]. IL-6 expression was found to be influenced by low HDL-c, and positively associated with disease progression in a cohort of WD GEP-NEN [46]. In vitro experiments with neuroendocrine pancreatic and midgut cells also showed the anti-proliferative effects of statins, pointing to a direct anti-tumor effect besides the lipid-lowering action [40,47].

Our study has some strengths and limitations. One of the strengths is having collected all data at diagnosis, overcoming any biases related to the effect of treatment on glycemic profile and body weight. The effect of abdominal obesity independently of BMI on disease prognosis was also evaluated. The limitations include the fact that this study was conducted in a relatively small patient cohort (*n* = 81). The majority of patients with MetS were under anti-hypertensive and statins drugs, which biased the evaluation of blood pressure, as well as lipid levels as RFs for WD GEP-NEN, especially for total cholesterol, LDL-c and HDL-c. The small number of patients under metformin treatment did not allow analyzing the influence of that antidiabetic drug on WD GEP-NEN outcomes. Another limitation is that the influence of the different tumor and metabolic targeted treatments on patient outcomes were not evaluated, nor were BMI trajectory and metabolic parameters within the follow-up.

## 5. Conclusions

In summary, a new era for oncological diseases is emerging, where lifestyle-related modifiable risk factors for cancer play an important role not only in the pathogenesis and incidence, but also in prognosis. Neuroendocrine neoplasia burden in the last four decades is likely to be part of this problem. In this study, we demonstrate that waist circumference, a surrogate measure for visceral obesity, is associated with significantly shorter PFS in WD GEP-NEN. This finding reinforces the potential link between visceral obesity and GEP-NEN, and further suggests that obesity could influence disease prognosis.

## Figures and Tables

**Table 1:** Patients and well-differentiated gastro-entero-pancreatic neuroendocrine neoplasia (WD GEP-NEN) tumor characteristics (*n* = 81), according to the presence of metabolic syndrome (MetS) criteria.

WD GEP-NEN	All Patients (*n* = 81)	Without MetS (*n* = 33)	With MetS (*n* = 48)	*p*-Value
Gender (Male)	41 (50.6%)	14 (42.4%)	27 (56.2%)	0.262
Age at Diagnosis (years)	60 (29–86)	53 (29–74)	63 (39–86)	0.002
Weight (kg)	71.2 (44.5–107.0)	67 (45.0–94.0)	75.0 (44.5–107.0)	0.005
BMI (kg/m^2^)	26.4 (20.1–36.9)	24.2 (20.1–36.3)	27.7 (20.5–36.9)	0.006
WC (cm)	96.0 (60.0–120.0)	87.0 (60.0–114.0)	100.0 (73.0–120.0)	<0.001
SBP (mmHg)	131.5 (98.0–214.0)	129.0 (98.0–152.0)	136.0 (104.0–214.0)	0.039
DBP (mmHg)	75.5 (46.0–116.0)	74.0 (47.0–95.0)	77.0 (46.0–116.0)	0.331
Total Cholesterol (mg/dL)	186.5 (107.0–333.0)	186.0 (109.0–288.0)	186.5 (107.0–333.0)	0.694
Calculated LDL-c (mg/dL)	103.6 (36.8–195.8)	104.9 (62.0–195.8)	102.3 (36.8–185.4)	0.93
HDL-c (mg/dL)	49.0 (21.0–92.0)	53.0 (31.0–92.0)	48.0 (21.0–69.0)	0.025
Triglycerides (mg/dL)	121.0 (4.0–627.0)	96.0 (48.0–254.0)	142.0 (59.0–627.0)	0.002
FPG (mg/dL)	101.0 (72.0–285.0)	91.0 (79.0–126.0)	110.0 (72.0–285.0)	<0.001
BMI Classification				
Normal weight	32 (39.5%)	21 (63.6%)	11 (22.9%)	0.001
Overweight	33 (40.7%)	6 (18.2%)	27 (56.2%)	
Obesity	15 (18.5%)	6 (18.2%)	9 (18.8%)	
Unknown	1 (1.2%)	0 (0.0%)	1 (2.1%)	
Family History of T2D	28 (34.6%)	12 (36.4%)	16 (33.3%)	0.802
T2D Classification (*n* = 81)				
Euglycemia	46 (56.8%)	26 (78.8%)	20 (41.7%)	0.002
FGA	16 (19.8%)	2 (6.1%)	14 (29.2%)	
T2D	19 (23.5%)	5 (15.2%)	14 (29.2%)	
MetS	48 (59.3%)	0 (0.0%)	48.0 (100%)	
MetS-WC	37 (45.7%)	7 (21.2%)	30 (62.5%)	<0.001
MetS-Hypertension	55 (67.9%)	12 (36.4%)	43 (89.6%)	<0.001
MetS-TG	32 (39.5%)	5 (15.2%)	27 (56.2%)	<0.001
MetS-HDL-c	46 (56.8%)	10 (30.3%)	36 (75.0%)	<0.001
MetS-FPG	46 (56.8%)	8 (24.2%)	38 (79.2%)	<0.001
Primary Tumor Site				
GI-NEN	66 (81.5%)	27 (81.8%)	39 (81.2%)	1
pNEN	14 (17.3%)	6 (18.2%)	8 (16.7%)	
Unknown	1 (1.2%)	0 (0.0%)	1 (2.1%)	
Hormonal Syndrome				
Functioning *	49 (60.5%)	16 (48.5%)	33 (68.8%)	0.202
Non-Functioning	24 (29.6%)	12 (36.4%)	12 (25.0%)	
Unknown	8 (9.9%)	5 (15.2%)	3 (6.2%)	
2010 WHO Grading ^#^				
NETG1	48 (59.3%)	17 (51.5%)	31 (64.6%)	0.241
NETG2	30 (37.0%)	15 (45.5%)	15 (31.2%)	
Unknown	3 (3.7%)	1 (3.0%)	2 (4.2%)	
Stage				
Locoregional Disease	26 (32.1%)	15 (45.5%)	11 (22.9%)	0.052
Metastatic Disease ^»^	55 (67.9%)	18 (54.5%)	37 (77.1%)	
First Treatment				
Somatostatin Analogues	13 (16.0%)	0 (0.0%)	13 (27.1%)	0.001
Surgery	63 (77.8%)	31 (93.9%)	32 (66.7%)	
TAE	3 (3.7%)	1 (3.0%)	2 (4.2%)	
Endoscopic Therapy	2 (2.5%)	1 (3.0%)	1 (2.1%)	
Hypertension Treatment	41 (78.9%)	8 (72.7%)	33 (80.5%)	0.669
Dyslipidemia Treatment Statins	25 (53.2%)	4 (36.4%)	21 (58.3%)	0.373
Fibrates	1(2.1%)	0 (0.0%)	1 (2.8%)	
T2D Treatment				0.2
Metformin ^ϒ^	7 (38.9%)	0 (0.0%)	7 (50%)	
Sulphonilurea	2 (11.1%)	1 (25.0%)	1 (7.1%)	
Other	3 (16.7%)	1 (25%)	2 (14.3%)	
Patient Outcomes				
Alive	58 (71.6%)	23 (69.7%)	35 (72.9%)	0.954
Death attributed to NEN	18 (22.2%)	8 (24.2%)	10 (20.8%)	
Death due to other causes	4 (4.9%)	2 (6.1%)	2 (4.2%)	
Lost to Follow-up	1 (1.2%)	0 (0.0%)	1 (2.1%)	
OS follow-up (months)	95.0 (16.8–262.5)	101.7 (16.8–206.8)	84.7 (17.3–262.5)	0.204
PFS follow-up (months)	50.6 (4.9–206.8)	50.6 (4.9–206.8)	50.7 (5.5–189.1)	0.966

WD GEP-NEN: well-differentiated gastro-entero-pancreatic neuroendocrine neoplasia; MetS: metabolic syndrome; BMI: body mass index; WC: waist circumference; SBP: systolic blood pressure; DBP: diastolic blood pressure; FPG: fasting plasma glucose; T2D: type 2 Diabetes Mellitus; FGA: Fasting Glucose Abnormalities; TG: Triglycerides; GI-NEN: gastrointestinal neuroendocrine Neoplasia; pNEN: pancreatic neuroendocrine neoplasia; WHO: World Health Organization; TAE: trans-Arterial Embolization; OS: overall survival; PFS: progression-free survival. * 44/49 patients with carcinoid syndrome (29 patients with MetS and 15 patients without MetS) and 3/49 (1.7%) patients with sporadic gastrinoma (100% with MetS). ^#^ WHO 2010 Grade was used to 2013, date of first patient enrolment, ^»^ 45/55 liver metastasis; 7/55 bone metastasis: 27/55 non-regional lymph node metastasis; 12/55 peritoneal implants; and 2/55 other locations. ^ϒ^ Metformin alone or associated with DPP-IV or SGLT2 inhibitors.

**Table 2:** WD GEP-NEN Mortality Overall Survival and Progression-free Survival at 5 and 10 years.

Characteristics	Years	OS		PFS	
		%	95% CI	%	95% CI
Metabolic syndrome					
No	5	90.9	74.4–97.0	40	21.3–58.1
	10	76.4	53.6–89.0	24.4	9.0–43.7
Yes	5	87.1	73.6–94.0	45.9	30.8–59.8
	10	72.5	55.3–84.0	18.1	7.0–33.5
Metabolic syndrome—Waist circumference					
No	5	90.9	74.4–97.0	48.3	29.5–64.8
	10	76.4	53.6–89.0	25.7	10.4–44.2
Yes	5	87.1	73.6–94.0	41.5	24.6–57.7
	10	72.5	55.3–84.0	17.6	6.6–32.9
Metabolic syndrome—Hypertension					
No	5	92.3	72.6–98.0	42.9	21.9–62.3
	10	86.9	64.0–95.7	32.1	13.7–52.3
Yes	5	87	74.6–93.6	44.2	29.9–57.6
	10	66.5	48.9–79.2	15.5	5.7–29.7
Metabolic syndrome—High Triglycerides					
No	5	89.7	77.1–95.6	47.3	31.3–61.8
	10	72	54.4–83.8	25.1	11.9–40.7
Yes	5	87.1	69.2–95.0	38.3	21.1–55.2
	10	77.2	55.1–89.4	13.4	2.8–32.3
Metabolic syndrome—Low HDL–c					
No	5	91.4	75.7–97.2	41.4	23.7–58.3
	10	77.7	56.0–89.6	30.7	13.9–49.3
Yes	5	86.6	72.6–93.8	45.3	29.6–59.9
	10	70.7	52.2–83.1	15.1	5.4–29.5
Metabolic syndrome—Fasting plasma glucose					
No	5	91.4	75.7–97.2	51.7	31.7–68.5
	10	77.1	54.2–89.5	27.9	11.6–47.0
Yes	5	86.5	72.5–93.7	38.3	23.8–52.7
	10	71.3	53.5–83.2	15.5	5.1–30.9

OS: Overall survival; PFS: Progression-free Survival.

**Table 3:** Hazard Ratios and 95% confidence intervals for Overall Survival and Progression-free Survival, according to patient and tumor characteristics, using univariate and multivariate logistic regression.

Characteristics	Overall Survival		Progression-Free Survival			
	Univariable		Univariable		Multivariable	
	HR	95% CI	HR	95% CI	HR	95% CI
Gender						
Male	1		1			
Female	0.89	0.38–2.06	0.79	0.46–1.36		
Age at Diagnosis (years)	1.02	0.97–1.06	0.99	0.97–1.02		
Primary Tumor Site						
GI-NEN	1		1			
pNEN	1.67	0.61–4.60	1.76	0.90–3.43		
Hormonal Syndrome						
Non-Functioning	1		1			
Functioning	1.14	0.46–2.85	1.2	0.66–2.20		
2010 WHO Grading						
NETG1	1		1		1	
NETG2	3.84	1.41–10.50	2.34	1.30–4.22	2.17	1.15–4.11
Stage						
Locoregional Disease	1		1			
Metastatic Disease	3.6	0.84–15.51	3.11	1.33–7.30		
BMI category						
Normal weight	1		1			
Overweight	0.8	0.30–2.11	1.2	0.65–2.24		
Obesity	1.29	0.43–3.85	1.27	0.59–2.72		
Diabetes Classification						
Euglycemia	1		1			
FGA	1.26	0.44–3.58	1.23	0.62.2.43		
T2D	0.58	0.19–1.81	1.26	0.66–2.42		
Weight	1.02	0.99–1.05	1.02	0.99–1.04		
BMI	0.99	0.89–1.11	1.02	0.95–1.09		
Waist Circumference	1.01	0.97–1.06	1.03	1.01–1.06	1.03	1.01–1.06
Systolic Blood Pressure	1	0.98–1.02	1.01	0.99–1.02		
Diastolic Blood Pressure	1.03	0.99–1.07	1.02	0.99–1.04		
HDL-c	0.97	0.94–1.01	0.99	0.97–1.02		
Triglycerides	0.99	0.98–1.01	1	0.99–1.01		
Fasting Plasma Glucose	0.99	0.97–1.01	1.02	0.99–1.03		
Metabolic Syndrome						
No	1		1			
Yes	1.01	0.43–2.35	1.03	0.59–1.83		
MetS-WC						
No	1		1			
Yes	1.15	0.45–2.93	1.39	0.78–2.49		
MetS-Hypertension						
No	1		1			
Yes	2	0.73–5.48	1.18	0.65–2.15		
MetS-HDL-c						
No	1		1			
Yes	1.04	0.43–2.48	1.28	0.73–2.25		
MetS-TG						
No	1		1			
Yes	1	0.39–2.52	1.21	0.70–2.09		
MetS-FPG						
No	1		1			
Yes	1.2	0.51–2.81	1.47	0.83–2.60		

HR: Hazard Ratio; 95% CI: 95% Confidence Interval; GI-NEN: gastrointestinal neuroendocrine neoplasia; pNEN: pancreatic neuroendocrine neoplasia; WHO: World Health Organization; FGA: Fasting Glucose Abnormalities; T2D: type 2 Diabetes Mellitus; BMI: body mass index; MetS: metabolic syndrome; WC: waist circumference; TG: Triglycerides; FPG: Fasting Plasma Glucose.

## Data Availability

The authors will make available the anonymized data used in this work upon request.
